# Cholelithiasis and Related Morbidity in Chronic Intestinal Failure: a Longitudinal Cohort Study from a National Specialized Centre

**DOI:** 10.1007/s11605-018-3979-3

**Published:** 2018-10-15

**Authors:** Nathan D. Appleton, Simon Lal, Gordon L. Carlson, Simon Shaw, Philip Stevens, Ioannis Peristerakis, Mattias Soop

**Affiliations:** Irving National Intestinal Failure Unit and The University of Manchester, Manchester Academic Health Science Centre, Salford Royal NHS Foundation Trust, Manchester, M6 8HD UK

**Keywords:** Intestinal failure, Parenteral nutrition, Cholelithiasis, Surgery, Cholecystectomy

## Abstract

**Background:**

Short-term studies have shown that patients with type III intestinal failure often develop gallstones and have recommended prophylactic cholecystectomy. In this retrospective cohort study, we aimed to define the incidence and clinical consequences of cholelithiasis over an extended time period, in order to refine the role of prophylactic cholecystectomy in type III intestinal failure.

**Methods:**

Data were retrospectively collected from a prospectively maintained audit. Patients with intestinal failure for 5 years or more were included. Kaplan-Meier analysis was used to estimate cumulative incidence over time. Predictors of cholelithiasis were evaluated by Cox regression.

**Results:**

Between 1 January 1983 and 1 December 2008, 81 patients were commenced on parenteral support lasting 5 years or more. Of 63 patients with no pre-existing gallstones on imaging, 17 (27%) developed gallstones during a median observation period of 133 months. On Kaplan-Meier analysis, the incidence at 10 years was 21%; at 20 years, 38%; and at 30 years, 47%. Thirteen of the 17 had symptoms and ten required surgical and/or endoscopic intervention. Increased weekly calorific content (P 0.003) and the provision of parenteral lipids (P 0.003) were predictors of cholelithiasis on univariable Cox regression.

**Conclusion:**

Many patients with long-term intestinal failure develop gallstones over time, with a 20-year incidence of 38%. The majority of those have symptoms or complications and require intervention. Therefore, prophylactic *en-passant* cholecystectomy is justified when gallstones are present in type III intestinal failure, supporting routine pre-operative imaging of the gallbladder prior to abdominal surgery.

## Introduction

The prevalence of cholelithiasis in the UK adult population is between 10 and 15%, with the majority of patients being asymptomatic.^1^ Patients with intestinal failure (IF) are at particular risk of developing gallstones due to altered gut anatomy and physiology.^2^ Intestinal failure is defined as a reduction of gut function below the minimum necessary for the absorption of macronutrients and/or water and electrolytes, such that intravenous support is required to maintain health.^3^ Long-term (type III) IF currently affects approximately 40 adults per million, or around 2600 adults in the UK.^4^

Because of the perceived increase in gallstone-related morbidity in type III IF, prophylactic cholecystectomy has been advocated. Some authors have recommended *en-passant* cholecystectomy during abdominal surgery in patients with IF and gallstones^5^ or even in all IF patients, irrespective of whether or not they have gallstones.^6,7^ Some authors have gone even further, to advocate undertaking a separate, prophylactic cholecystectomy in patients who are found to have developed gallstones during ultrasound surveillance^6^ or even in all patients with type III IF.^8^ These recommendations were based upon cohort studies undertaken in the 1980s and 1990s, when hyperalimentation was widely used,^6,9,10^ and substantial morbidity and mortality after cholecystectomy for gallstone-related illnesses were reported.^7,8,10^ These historical studies were mainly cross-sectional and limited in duration of intestinal failure to 14–39 months, and it is therefore been unclear to what extent they are applicable to the current population of patients requiring long-term parenteral support for IF. Noting the paucity of contemporary data, the European Society for Clinical Metabolism and Nutrition (ESPEN) have recently taken a more conservative approach, recommending that gallstones in type III IF be managed no differently from that in the general population, with cholecystectomy reserved for only for those with symptomatic or complicated gallstone disease.^2^

The aim of the current study was to provide comprehensive, longitudinal, and long-term data on the incidence of symptomatic and asymptomatic cholelithiasis and gallstone-related intervention and complications in a cohort of patients with type III IF, in order to define the incidence of gallstone disease and provide a more rational evidence base to support decisions regarding the role of prophylactic cholecystectomy in this patient group.

## Materials and Methods

### Study Design

This is a retrospective study of a prospectively maintained audit database of all patients with intestinal failure managed at the Irving National Intestinal Failure Unit at Salford Royal Hospital, Manchester, UK.

### Participants

All patients with type III IF commenced on home parenteral support between 1 January 1983 and 1 December 2008 and who received this support for 5 years or more were included in this study. Patients with a documented diagnosis of gallstones or cholecystectomy prior to commencing home parenteral support were excluded. The STROBE guidelines for cohort studies were followed (www.strobe-statement.org).

### Outcomes

The study cohort was studied longitudinally. Data recorded included patient demographics, underlying IF diagnosis, date of commencement of parenteral support, parenteral support prescription (weekly volumes, calorific content, and fat provision), length of small bowel in continuity, and whether or not the colon was in continuity. The weekly provision of parenteral calories was defined as low (0–500 kcal/week), medium (500–13,000 kcal/week), or high (< 13,000 kcal/week). A diagnosis of cholelithiasis at any point during the period of parenteral support was recorded, along with the modality and the indication for investigation that resulted in the diagnosis (symptoms suggestive of cholelithiasis, deranged liver function tests, or incidental finding). Types of complication of cholelithiasis and their consequent management were recorded. All patients were reviewed in the intestinal failure outpatient clinic at Salford Royal Hospital at least every 6 months during the study period. Data were censored at the time of study, when parenteral support was discontinued or when patients died, whichever occurred first. Hence, any biliary disease diagnosed after the period of nutritional support was intentionally excluded, and the periods of nutritional support and data collection were identical.

### Statistical Methods

A Kaplan-Meier curve was generated for the incidence of cholelithiasis. Simple and multivariable Cox regression analyses were performed to identify factors associated with development of gallstones (JMP 13.0 for Mac OS X, SAS, Cary, NC, USA). Where data were missing for variables, analyses were undertaken with data available and the number of missing data points was indicated.

## Results

### Patient Characteristics

Between 1 January 1983 and 1 December 2008, 81 patients commenced parenteral support for at least 5 years. All patients had undergone ultrasound and cross sectional abdominal imaging prior to commencing parenteral support. Sixteen patients were excluded because these investigations already showed gallstones or confirmed previous cholecystectomy. A further two patients with no identifiable history of cholecystectomy were also excluded as their imaging showed no evidence of a gallbladder and these patients were therefore assumed to have had previous cholecystectomy. This left a study cohort of 63 patients (20 males, 43 females) in whom there was no evidence of pre-existing cholelithiasis.

Data were censored on 19 December 2013. While there was no loss to follow-up in the study cohort, some data were missing regarding BMI (*n* = 15) and small bowel length (*n* = 13), and whether the colon was present and in continuity (*n* = 11).

Median (range) age at the commencement of parenteral nutrition was 43 (18–78) years and the median (range) duration at the time of data collection of 133 (60–365) months. Indications for parenteral support are outlined in Table 1. Ten of 50 patients with known small bowel length (20%) had < 50 cm of small bowel remaining and 44 of 52 patients with data on the presence of the colon (85%) had their colon in circuit.

**Table 1:** Indication for home parenteral support

Indications	Number (%)
Crohn’s disease	22 (35)
Mesenteric ischemia	12 (19)
Motility disorder	12 (19)
Postoperative complication*	6 (10)
Radiation enteritis	5 (8)
Scleroderma	3 (5)
Volvulus	2 (3)
Familial adenomatous polyposis	1 (2)

^*^Surgery for Crohn’s disease excluded

The median (range) number of cyclical nocturnal infusions was 6 (2–7) per week. Thirty-three patients (52%) had low, 22 (35%) medium, and 8 (13%) high calorific provision. Nine (14%) patients had glucose provision as the sole energy source, 23 (36%) received lipids one night per week; 15 (24%), two nights; 2 (3%), three nights; 1 (2%) four; and 4 (6%) received lipids five nights per week. Nine patients (14%) received electrolyte only bags with no lipid or glucose content.

### Cholelithiasis During Home Parenteral Support

**Fig. 1:**
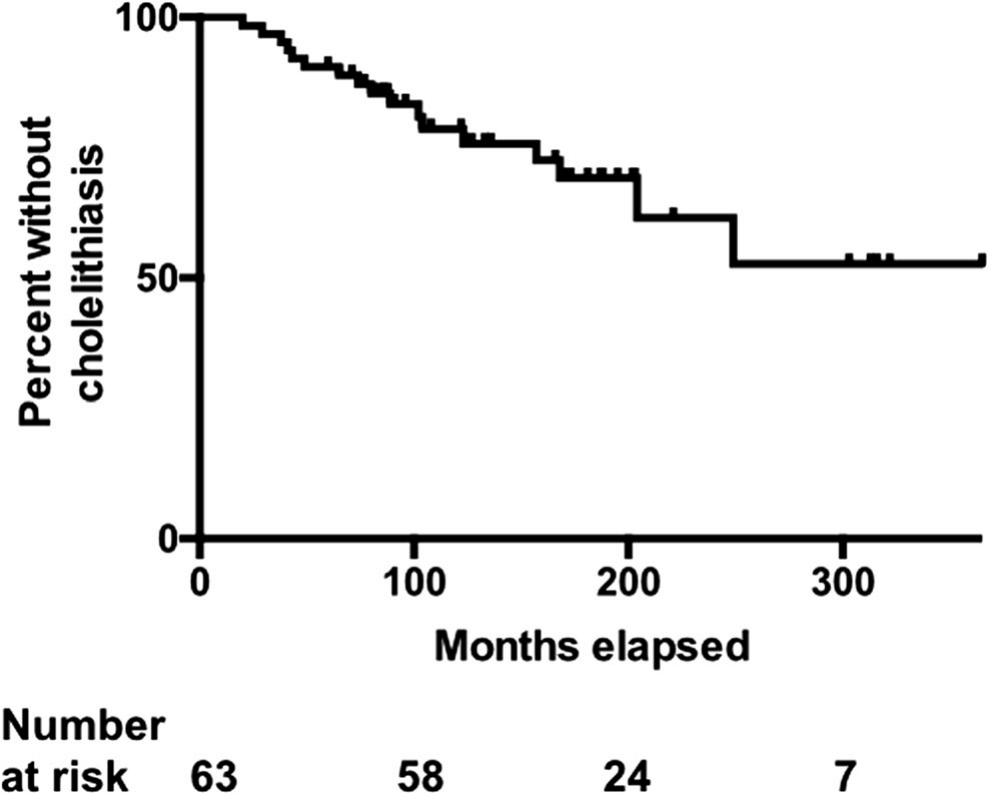
Kaplan-Meier plot showing patients without cholelithiasis over 365 months of home parenteral support

Seventeen of the 63 patients (27%) developed gallstones during the median observation period of 133 (60–365) months. By Kaplan-Meier analysis, the incidence of gallstones was 21% over an observation period of 10 years; 38% after 20 years; and 47% after 30 years (Fig. 1).

Diagnosis of gallstones was made by ultrasound scan in 13, computerized tomography in three and magnetic resonance cholangiopancreatography (MRCP) in one patient. Six patients had a MRCP subsequent to diagnosis of cholelithiasis to guide treatment. Indications for the investigations were symptoms suggestive of cholelithiasis in 10 patients and deranged liver function tests in 4 otherwise asymptomatic patients. In three patients, cholelithiasis was an entirely incidental finding during follow-up.

### Complications and Management of Cholelithiasis

During the study period, 13 of the 17 patients with gallstones developed symptoms, complications, or both. Four patients presented with biliary colic, four with acute pancreatitis, two with common bile duct stones, one with acute uncomplicated cholecystitis, one with cholangitis, and one with a gallbladder empyema and an associated liver abscess.

Of the 17 patients who developed gallstones, seven were conservatively treated, while ten required treatment. Five patients underwent an elective open cholecystectomy, two had an elective laparoscopic cholecystectomy and one required an emergency laparotomy for a surgical cholecystostomy and drainage of a liver abscess. Two patients underwent endoscopic retrograde cholangiopancreatography (ERCP) as their definitive procedure; one patient had ERCP prior to elective cholecystectomy. The patient who required a laparotomy had permanent biliary drainage via a cholecystostomy. No complications occurred as a result of either endoscopic or surgical interventions.

### Predictors of Cholelithiasis

Simple Cox regression analyses exploring associations between possible predictors and the occurrence of gallstones are presented in Table 2. Increased weekly calorific provision and the parenteral administration of lipids were found to be associated with cholelithiasis; while gender, age, year at commencement, BMI, and intestinal anatomy were not.

**Table 2:** Simple Cox regression models evaluating univariable associations between putative risk factors of cholelithiasis in type III intestinal failure

Independent variable	*P* value	Effect size
Gender	0.170	–
Age at start of parenteral nutrition (years)	0.890	–
Calendar year at start of parenteral nutrition	0.188	–
BMI (kg/m^2^)	0.365	–
Small bowel length (cm)	0.608	–
Colon in continuity (yes/no)	0.328	–
Parenteral calories (kcal/week)	0.003	1.000135*
Parenteral lipids (yes/no)	0.003	9.34#

*parameter estimate

#risk ratio (lipid vs no lipid provision)

Due to the limited number of events (diagnoses of gallstones), multivariable Cox regression was performed using only the two strongest predictors on univariable analysis. In this model (*P* = 0.002), neither calorific content (*P* = 0.073) nor the provision of lipids (*P* = 0.088) were independent predictors of gallstone development.

## Discussion

The present study provides the first data from a longitudinal study of an at risk population on the development of gallstones and the need for treatment of gallstone-related morbidity in patients receiving parenteral support for 5 years or more. The results confirm that the risk of forming gallstones markedly exceeds that usually expected in the general population, with a 20-year incidence of new gallstone disease of 38%.

Not only was cholelithiasis found to be frequent in type III intestinal failure, but the majority of patients who developed gallstones in the present study had clinical manifestations of their disease. Three-quarters of patients had symptoms or complications, with 59% requiring surgical or endoscopic intervention during the median observation period of 133 months. In contrast, of the 10–15% of people who have gallstones in the general population in England, only 1–4% develop symptoms and fewer than 1% have been said to require intervention each year.^11^

These results expand on those of previous reports on the incidence and prevalence of cholelithiasis in type III IF. These earlier studies were undertaken during an era when intravenous hyperalimentation was a common practice, were usually cross-sectional in nature and, consequently and most importantly, did not exclude patients with pre-existing gallstones. This may explain why the reported incidence of gallstones has been said to be as high as 23–100% over a mean duration of parenteral nutrition of only 14–39 months in these earlier studies.^5–10^ In contrast, we followed up patients longitudinally for much longer in the present study and their intravenous regimens were consistent with current practice including a reduction in the amount of soybean-based lipid routinely administered.^2^ The present data probably therefore represent a more accurate assessment of the likelihood that a patient with type III IF will develop gallstones during the course of their treatment and require intervention for gallstone-related morbidity, and might be used as the basis of formulating future treatment policy.

In the present study, higher calorific content and lipid provision were associated with cholelithiasis on univariable analysis, while gender, BMI, and underlying bowel anatomy were not. Dray et al. also studied putative predictors and found that having no or negligible oral intake (as opposed to a limited or normal oral diet) was the main contributing factor.^5^ Unfortunately, our database did not allow us to collect data on oral dietary intake and, in any event, the proportion of energy absorbed could not have been estimated, but our finding that higher parenteral caloric requirements were associated with cholelithiasis is consistent with this observation. Dray and colleagues also found no differences for gender and BMI and, contrary to the present findings, they found no effect of fat provision.

Multiple mechanisms for gallstone formation in parenteral nutrition patients have been suggested. They include prolonged periods of starvation, provision of lipid, ileal disorders including Crohn’s disease, absence of ileum or ileocecal valve, rapid weight loss, and medication such as opiates and anti-cholinergics.^12^ During prolonged starvation, there is modification of normal bile acidification and a reduction in the secretion of cholecystokinin.^13^ Gallbladder contractility is consequently reduced, promoting biliary stasis and the formation of biliary sludge.^14^ Interruption of the enterohepatic circulation and loss of bile salts following ileal resection leads to cholesterol supersaturation and sludge formation.^15^ Additionally, biliary bilirubin concentrations in patients with terminal ileal Crohn’s disease have been found to be increased two to threefold, leading to a potentially higher risk of pigment stone formation.^16^ Current recommendations for prevention of cholelithiasis in IF include allowing oral nutrition as tolerated and limiting use of narcotics and anticholinergics.^2^ Based on the present findings, judicious usage of lipids may also have a role.

The present results have implications for the role of prophylactic cholecystectomy in type III IF. While previous studies uniformly recommended prophylactic cholecystectomy,^5–10^ current recommendations from ESPEN have taken the opposing view, advising that intervention be reserved for symptomatic or complicated gallstone disease only.^2^ Addressing first the role of prophylactic surgery in any patient with type III IF and an apparently healthy gallbladder, the current data show that although a significant proportion of patients with type III IF will eventually develop gallstones, the majority will not. Furthermore, when intervention is required to manage gallstones, our data suggests that this is safe, and the high morbidity and mortality reported by early studies were not observed presently. The practice of routinely undertaking prophylactic cholecystectomy, either as a sole procedure or *en-passant* during abdominal surgery undertaken for other reasons, in patients with type III IF but without gallstones, is therefore not supported by the present findings.

On the other hand, our results show that once gallstones do develop, the majority of patients with type III IF, unlike individuals without IF, will experience symptoms or complications, and most will need intervention. A current biliary ultrasound scan is therefore a prudent addition to the work-up in preparation for elective laparotomy in patients with type III IF. *En-passant* cholecystectomy during abdominal surgery in patients with type III IF known to have gallstones therefore appears to be a reasonable recommendation, provided this additional procedure is anticipated to have a low risk of morbidity. *En-passant* prophylactic cholecystectomy may be associated with risks of bleeding and bile leakage, which may considerably increase the risk of the other procedures for which laparotomy is intended in this patient group so the relevant preoperative discussions should carefully reflect the balance of risk and benefit.

While the present study is based on a clinical audit database, prospectively maintained in a national intestinal failure unit over several decades, it has several significant limitations. Even in a national center, the number of patients available for follow-up over such a long period is only modest as is inevitable with uncommon conditions such as this. One previous study included more patients (*n* = 119), but they were followed for a median period of 15 months only.^5^ With such a short duration of treatment, weaning off parenteral support would have been anticipated in a significant proportion and the data probably do not reflect the long term risks of gallstone development as indicated in the present study. While detailed and regular follow-up of a large cohort of patients receiving long term parenteral support was a key strength of the present study, the patients did not undergo formal planned surveillance with regular ultrasound scans, only cross sectional imaging or ultrasound as required for clinical reasons and it possible that, although the percentage of patients who developed gallstones in the present study was high, our figures might still represent an underestimate.

## Conclusion

In conclusion, patients with IF who receive parenteral support for more than 5 years are increasingly susceptible to cholelithiasis over time, so that after 20 years, 38% of patients have developed gallstones. Risk factors include higher rates of calorific and parenteral lipid provision. Three-quarters of patients who developed gallstones develop symptoms or complications of them and most required intervention. While prophylactic cholecystectomy would appear unjustifiable in these patients, *en-passant* cholecystectomy during abdominal surgery undertaken for other reasons (for example reconstructive abdominal surgery) would appear to be appropriate in patients who have asymptomatic gallstones.

### Summary of Author Contributions

**Table:** 

	NA	SL	GC	SS	PS	IP	MS
Substantial contributions to conception or design		X	X		X		X
Acquisition of data	X			X	X	X	
Analysis of data	X					X	X
Interpretation of the data	X	X	X				X
Drafting or revising the work	X	X	X	X	X	X	X
Final approval of work	X	X	X	X	X	X	X
Agreement to be accountable for all aspects of the work	X	X	X	X	X	X	X
